# Functional outcomes may vary over time after patellar tendon and knee intra-articular heterotopic ossification excision: A case report^☆^

**DOI:** 10.1016/j.ijscr.2024.110773

**Published:** 2024-12-31

**Authors:** Mohammad Ayati Firoozabadi, Hesan Rezaee, Mohammadreza Razzaghof, Seyed Mohammad Javad Mortazavi

**Affiliations:** a Joint Reconstruction Research Center, Tehran University of Medical Sciences, Tehran, Iran; b Department of Orthopedic Surgery, Imam Khomeini Hospital Complex, Tehran University of Medical Sciences, Tehran, Iran; c Joint Reconstruction Research Center (JRRC), Imam Khomeini Hospital, Tehran University of Medical Sciences, End of Keshavarz Blvd, 1419733141 Tehran, Iran

**Keywords:** Heterotopic ossification, Tibia nailing, Knee contracture, Surgical management, Physical therapy

## Abstract

**Introduction:**

Heterotopic ossification (HO) is the formation of mature bone in soft tissue, often occurring after fractures and trauma. Patients with HO experience pain, joint stiffness, and other complications. Treatment aims to improve function; surgical procedures have succeeded in 83.3 % of cases. Existing literature has extensively documented instances of HO occurring in the acetabulum, elbow, and during total hip arthroplasty (THA). HO formation is rare around the knee and intraarticularly after tibia nailing. A unique aspect of our case is the period of restricted ROM in the knee following surgical excision. Additionally, physical therapy played a crucial role in restoring full ROM after this period.

**Case:**

A 38-year-old man presented to our department with right knee pain and restricted ROM resulting from a tibia fracture nailing performed three years earlier in another hospital. Radiographic imaging revealed HO in the retro patellar and intraarticular areas. He underwent surgical excision to remove the HO but continued to experience limited ROM during the follow-up period. Despite undergoing various treatments, including chemoprophylaxis and under anesthesia manipulation, his ROM did not significantly improve. Eventually, after long-term physical therapy, his condition improved, and at the two-year follow-up, he was pain-free with enhanced ROM.

**Discussion:**

Surgical resection is recommended when HO significantly affects joint range of motion, but incomplete resection is linked to recurrence. After surgical removal of HO, prophylaxis with indomethacin or single fraction radiotherapy is used. However, limitations in range of motion may persist after surgical excision, and recurrence can occur despite preventive measures. Physical therapy may play a crucial role in restoring range of motion and achieving optimal treatment outcomes.

**Conclusion:**

Tibia nailing may contribute to the formation of HO and can lead to a restricted ROM in the knee. It is essential to diagnose HO early and consider a multidisciplinary approach that includes surgical excision, chemoprophylaxis, and physical therapy. The role of physical therapy might be more significant than previously thought.

## Highlights

• Heterotopic ossification may occur after reamed tibia nailing.
• HO treatment consists of surgical excision, NSAID, and low-dose radiotherapy.
• Physical therapy following surgery plays a crucial role in achieving optimal functional outcomes.
• Early intervention and long-term follow-up in the management of HO are crucial.
• The fluctuation of patient function during the recovery period is a crucial aspect to consider.

## 1 Introduction

Heterotopic ossification (HO) is the formation of mature lamellar bone in soft tissue where bone usually does not exist. Traumatic HO often occurs in the periarticular tissues following fractures and fracture-dislocations of the acetabulum, hip, elbow, knee, and shoulder and is associated with the presence of a traumatic brain injury, polytrauma, spinal cord injury, and an Injury Severity Score (ISS) ≥ 16 [1]. However, the formation of HO around the knee following tibial nailing is rare, with only a few case reports available [[2], [3], [4]]. Previous cases have involved complications such as brain damage or fat embolism. HO formation after tibial nailing, particularly in the absence of brain damage or spinal cord injury, is uncommon.

Patients with HO experience a wide range of problems; these include pain, joint range of motion loss, joint ankylosis, skin ulceration, overlying skin graft failure, muscle entrapment, neurovascular entrapment, and prosthetic limb fitting difficulties [5]. The main goal of treatment is the patient's functional improvement. Functional goals of surgical procedures were reached in 83.3 % of cases [6]. Previous studies have primarily focused on hip, elbow, and neurologic cases. Limited articles exist about HO formation after tibial nailing.

This article presents the surgical treatment of a case of heterotopic ossification of the patellar tendon after intramedullary nailing of an open tibia shaft fracture, problems in the follow-up period, and the patient's outcome. The work has been reported in line with the SCARE criteria [7]. The patients were informed about using the data for publication, and informed consent was obtained.

## 2 Case report

**Fig. 1:**
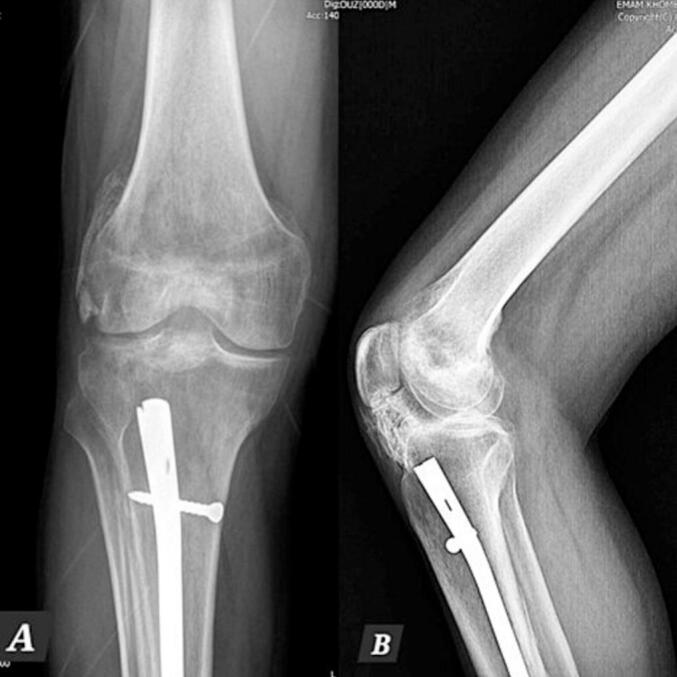
A and B: AP and Lateral views showed type 4 Brooker HO at the patellar tendon and type 2 at LCL.

**Fig. 2:**
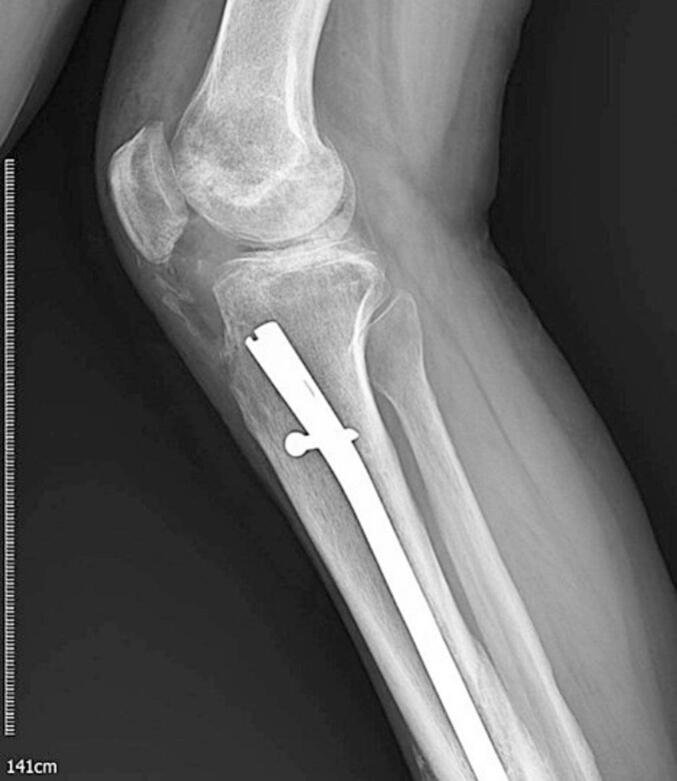
Early postoperative lateral view radiography demonstrates complete HO excision.

**Fig. 3:**
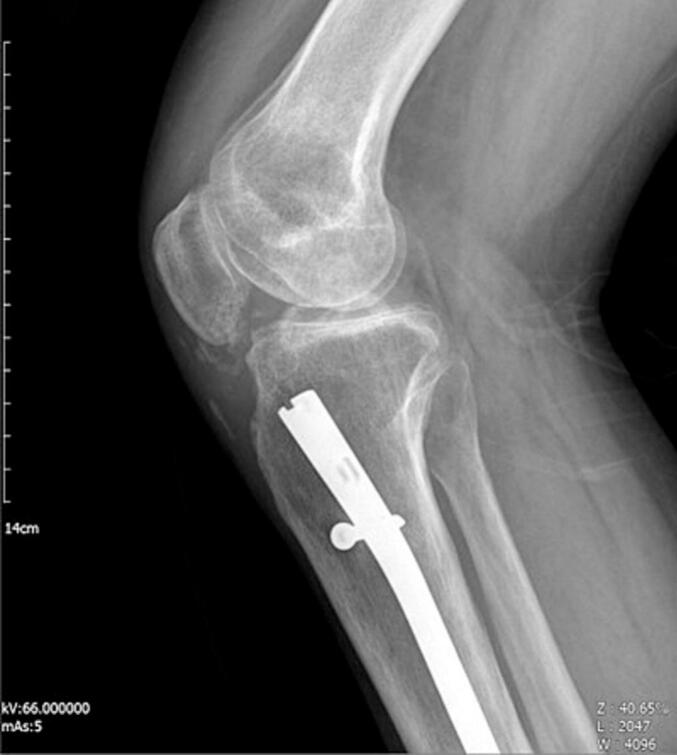
The lateral view demonstrates type 1 brooker HO recurrence after two month.

**Fig. 4:**
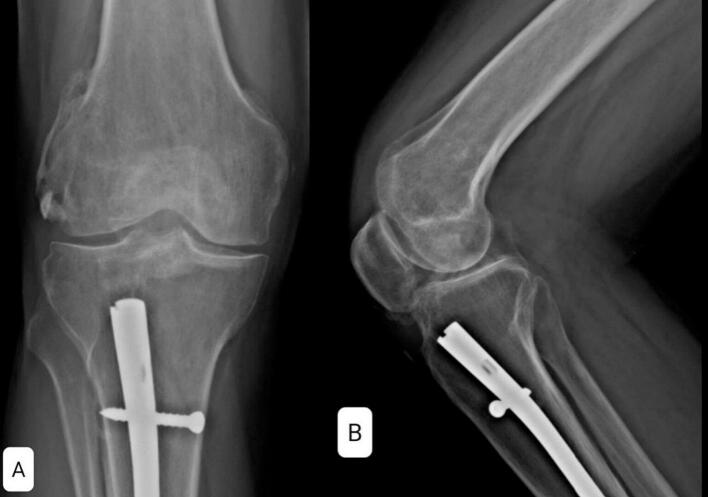
Knee radiography after two years shows heterotopic ossification in the lateral collateral ligament and retropatellar region, with some degree of patella baja. A: AP view B: Lateral view.

**Fig. 5:**
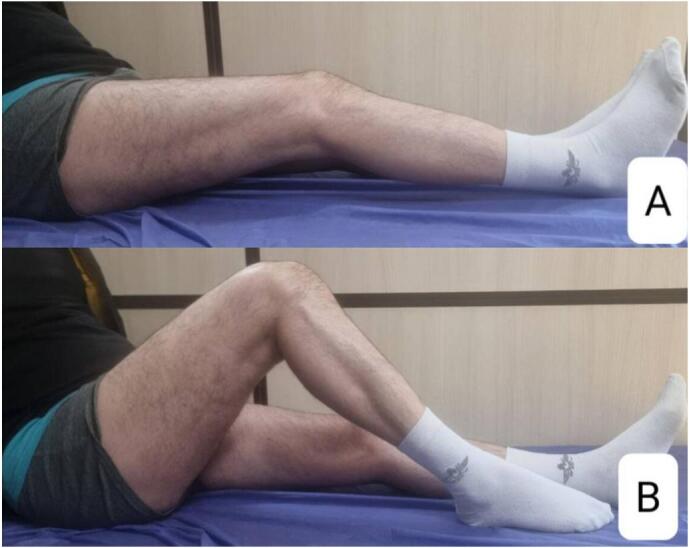
The photograph shows the ROM of the knee at the two-year follow-up. A: Extension B: Flexion.

A 38-year-old man presented at the clinic with complaints of restricted right knee ROM, pain, and popping. His symptoms began after a car accident three years ago. He did not suffer from any brain trauma or spinal cord injury. He was treated for a Gustilo type 2 open fracture of the tibia shaft, which involved irrigation and reamed nailing at another hospital. However, five months later, he started experiencing knee pain and a gradual reduction in knee range of motion (ROM), measured at 0–45-90 during the examination at our clinic. Radiographic imaging revealed Brooker type 4 HO in the patellar tendon and knee intra-articular and type 2 HO in the lateral collateral ligament (Fig. 1). The patient experienced a limited range of motion in his knee and pain, making it difficult to complete his daily tasks effectively. Due to mature HO and decreased range of motion, he underwent surgical excision of the HO through a medial parapatellar approach. We chose open arthrotomy due to the involvement of the retropatellar region, a large HO that affects the entire fat pad, and the risk of incomplete excision with the arthroscopic technique. There was an HO in the retropatellar area, extending from the tibial plateau to the patella, which was engaged with the femoral notch in extension. There were also small HOs in the lateral collateral ligament (LCL) region and around the anterior cruciate ligament (ACL) that were excised (Fig. 2). Following the surgery, his ROM improved to 0–10-120 (10 degrees flexion contracture and 120 degrees maximum flexion). He commenced physical therapy the day after the surgery and was discharged with a prescription for Indomethacin 25 mg three times a day for six weeks for HO prophylaxis. After two months of follow-up, the patient did not regularly complete their physiotherapy sessions. The patient reported reduced ROM (0-20-80), and radiography revealed new HO formation in the patellar tendon and intra-articular knee (Fig. 3). Following the limitation of knee ROM and interference with the patient's daily activities, Aggressive physical therapy was initiated but did not yield improvement after one month. As a result, the patient underwent manipulation under anesthesia to improve ROM. Despite aggressive post-manipulation physiotherapy, the knee's ROM was measured at 0–20-50 after four weeks. The patient made an informed decision to decline surgical intervention, opting instead for a regimen of long-term physical therapy (Two days per week, with each session lasting 90 min). This approach resulted in a gradual improvement in his ROM. At the two-year follow-up, the patient was pain-free, and his range of motion had improved to 0–20-100 degrees. Examination and radiography revealed patella baja (Fig. 4, Fig. 5).

## 3 Discussion

Heterotopic ossification was described as early as 1969 and has been documented in numerous areas of the body [8]. It can occur in individuals aged from infancy to late adulthood, with men being slightly more frequently affected than women. In most cases, trauma is the triggering event, with up to 75 % of cases being linked to trauma [9]. This can occur after traumatic or atraumatic injury. Acetabular fractures, antegrade femoral intramedullary nailing, elbow Fracture-dislocations, burns, and head trauma are traumatic injuries that can cause HO formation [4]. The exact pathophysiology of HO is still unknown, and many hypotheses have been proposed, such as the role of multipotent cells of mesenchymal origin and exaggerated inflammatory response [5].

Fractures of long bones can be treated with intramedullary nailing. There are reports about HO formation after femur fracture nailing. Markes et al. found a direct correlation between IM nailing of the femur, head injury, spinal cord injury, ventilator days, ICU days, and HO formation [10]. HO following tibial fracture nailing is a relatively uncommon. This case underscores the significance of actively screening for HO formation in patients who exhibit a restricted range of motion in the knee following tibial nailing procedures.

Table 1 demonstrates previous studies about HO formation after tibia nailing and its outcomes. The literature describes that the patient could be symptomatic six months to 3.5 years after the injury. The radiographic review revealed that tendinous calcification began to develop three months after nailing [4]. The patellar tendon was the most common site of HO formation, and the MCL was the second site [11]. HO could happen after all types of approaches are used for tibia nailing [2,4,11].

**Table 1:** Previous studies and their outcomes.

Kent et al. (2018) [11]	Twenty-six floating knees were treated with antegrade and retrograde femoral nailing and trans patellar tibia nailing. The patellar tendon was the most common site of HO formation, and the MCL was the second site.
Krishnan et al. (2016) [3]	A case of tibia shaft fracture with fat emboli was treated with trans patellar unreamed tibia nailing. HO formation was at six weeks post-operative in the retropatellar fat pad. Surgical excision and indomethacin prophylaxis were used for treatment. ROM was normal at six months f/u.
Howell et al. (2011) [4]	A case of open tibia shaft fracture was treated with irrigation and medial parapatellar reamed tibia nailing. The HO formation was 3.5 years post-injury and treated with surgical excision.
Rehman et al.(2019) [2]	A case of Gustilo IIIB tibial shaft fracture treated by suprapatellar tibial nailing. Two months later, Radiographs demonstrated an intra-articular bony fragment. The fragment was removed arthroscopically, and his symptoms were completely resolved.
Gosselin et al. (1993) [8]	Two cases of open tibia shaft fracture with intracranial hematoma were treated with trans patellar reamed tibia nailing. HO formation was at the patellar tendon.
Mitsionis et al. (2009) [16]	In 23 knees of 14 ICU patients who experienced HO within the knee, 82 % experienced increased ROM (19 knee joints), 93 % experienced improved sitting, and 57 % experienced improved ambulation following resection of heterotopic ossification.

No appropriate primary prophylaxis methods exist currently, but some methods are used more than others. Literature suggestions include non-steroidal anti-inflammatory Drugs (NSAIDs), radiotherapy, Bisphosphonates, and passive movement therapy [5]. Some experimental prophylaxis, like echinomycin antibiotics and local lipopolysaccharide injection, are under examination [5,12]. Saphira et al. study showed that NSAID prophylaxis for HO may have better efficacy than RT in high-risk patients [13]. We used Indomethacin as a prophylactic treatment after the surgical HO excision. However, a recurrence of HO has occurred. It is important to note that most studies on HO have focused on primary HO formation rather than recurrence. This area requires further investigation regarding the choice of prophylactic treatments during the recurrence phase.

Our study found no clear relationship between the severity of HO and knee ROM. This finding is consistent with the research conducted by Kent et al., which showed a poor correlation between the severity of HO and knee ROM following floating knee treatment using nailing [11]. However, When HO significantly affects the joint's ROM, surgical resection is recommended only after the HO fully matures, which may take 12–18 months. Some have advocated that excision should be performed early to prevent irreversible loss of motion; a comparison between 18 patients with elbow ankylosis and 27 patients with partial restriction of motion demonstrated a comparable return of motion [14]. HO excision can be performed using an arthroscopic or open surgical approach. Although complete excision is not always practical or possible, incomplete resection of the HO is associated with recurrence [9]. Agarwal et al. found that surgical excision of HO results in recurrence through similar mesenchymal cell populations and signaling mechanisms present in the initial developing HO lesion [15]. In our case, due to the retropatellar and intraarticular position of HO, we chose an open technique to reduce the chance of incomplete HO excision.

Previous studies showed that the functional goal of HO treatment could be reached in 83.3 % of patients [6]. Previous case reports of HO formation around the knee after tibial nailing showed good outcomes following surgical excision; however, they reported recurrences of HO, though there were no functional limitations [[2], [3], [4]]. Similar to the previous study, our case showed a good outcome with a recurrence of HO at the end. However, the patient experienced a period of restricted ROM. Long-term physical therapy was instrumental in helping him restore knee ROM. Consequently, it is essential to acknowledge physical therapy's significant role in treating HO.

## 4 Conclusion

In conclusion, tibia nailing may contribute to the formation of HO and can lead to a restricted ROM in the knee. It is important to diagnose HO early and consider a multidisciplinary approach that includes surgical excision, chemoprophylaxis, and physical therapy. The role of physical therapy might be more significant than previously thought; however, further research is necessary to determine its exact effectiveness in the treatment of HO.

## Patient consent

The patient provided written informed consent for publication and any accompanying images. The editor-in-chief of this journal can review a copy of the written consent upon request.

## Ethics approval and consent to participate

Ethical approval is exempt at our institution.

## Guarantor

SM Javad Mortazavi, MD

## Research registration number

N/A

## Consent for publication

Not applicable.

## Funding

There is no funding source.

## Author contribution

Mortazavi. MJ: Study concept and design

Ayati. M: supervision, Project Administration

Rezaee. H: Writing - Original Draft

Razzaghof. M: Investigation and Validation

## Conflict of interest statement

There are no conflicts of interest to declare. All co-authors have agreed with the final manuscript's contents, and no financial interest remains to be announced.

## Data Availability

Not applicable.
